# Patient-Derived Induced Pluripotent Stem Cell-Based Models in Parkinson’s Disease for Drug Identification

**DOI:** 10.3390/ijms21197113

**Published:** 2020-09-26

**Authors:** Georgia Kouroupi, Nasia Antoniou, Kanella Prodromidou, Era Taoufik, Rebecca Matsas

**Affiliations:** Laboratory of Cellular and Molecular Neurobiology-Stem Cells, Hellenic Pasteur Institute, 127 Vassilissis Sofias Avenue, 11521 Athens, Greece; gkouroupi@pasteur.gr (G.K.); nantoniou@pasteur.gr (N.A.); kprodromidou@pasteur.gr (K.P.); etaoufik@pasteur.gr (E.T.)

**Keywords:** disease phenotypes, high-content screening, chemical libraries, disease-modifying drugs, hiPSC-derived neurons, hiPSC-based co-culture systems, brain organoids

## Abstract

Parkinson’s disease (PD) is a common progressive neurodegenerative disorder characterized by loss of striatal-projecting dopaminergic neurons of the ventral forebrain, resulting in motor and cognitive deficits. Despite extensive efforts in understanding PD pathogenesis, no disease-modifying drugs exist. Recent advances in cell reprogramming technologies have facilitated the generation of patient-derived models for sporadic or familial PD and the identification of early, potentially triggering, pathological phenotypes while they provide amenable systems for drug discovery. Emerging developments highlight the enhanced potential of using more sophisticated cellular systems, including neuronal and glial co-cultures as well as three-dimensional systems that better simulate the human pathophysiology. In combination with high-throughput high-content screening technologies, these approaches open new perspectives for the identification of disease-modifying compounds. In this review, we discuss current advances and the challenges ahead in the use of patient-derived induced pluripotent stem cells for drug discovery in PD. We address new concepts implicating non-neuronal cells in disease pathogenesis and highlight the necessity for functional assays, such as calcium imaging and multi-electrode array recordings, to predict drug efficacy. Finally, we argue that artificial intelligence technologies will be pivotal for analysis of the large and complex data sets obtained, becoming game-changers in the process of drug discovery.

## 1. Introduction

Parkinson’s disease (PD) is the second most common neurodegenerative disorder, with an increasing incidence in aged people, while an estimated 4% are diagnosed before the age of 50. Due to the high prevalence of PD and the increase in the proportion of aging population resulting from extended life expectancy, the disease has become a rapidly growing area of concern. PD is characterized by progressive loss of striatal-projecting midbrain dopaminergic neurons of the substantia nigra pars compacta [1], leading to motor symptoms, such as bradykinesia, rigidity and resting tremor, with a wide range of severity [2]. Although considered a classic movement disorder, PD is associated with a broad spectrum of non-motor symptoms, including psychiatric and cognitive manifestations such as depression, dementia and hallucinosis, as well as sleep and sensory disturbances. These persistent symptoms may appear long before motor dysfunction becomes apparent while they remain unaffected by currently available therapeutics, severely impairing patients’ quality of life [3].

Most PD cases are sporadic with unknown etiology; however, an approximate 10% represent familial cases (reviewed in [4]). Among the genes linked to heritable monogenic PD, mutations in the α-synuclein gene *SNCA* (*PARK1/4*) [5] and the leucine-rich repeat kinase 2 gene *LRRK2* (*PARK8*) [6] are accountable for autosomal-dominant PD forms, while mutations in *PINK1* (PTEN-induced putative kinase 1; *PARK6*) [7], *Parkin* (*PARK2*) [8], protein deglycase *DJ-1* (*PARK7*) [9], *ATP13A2* (*PARK9*) [10] and β-glucocerebrosidase (*GBA*) [11] are responsible for an autosomal recessive mode of inheritance.

Currently, only symptomatic or palliative treatments are available with none capable to prevent or slow down PD progression and revert neurological disabilities. Regardless of the divergent pathologies and clinical manifestations, dopamine-replacement drugs such as Levodopa (L-dopa), which was identified 53 years ago, are used as a first-line treatment to address the cardinal motor issues arising from nigrostriatal degeneration [12]. For most patients though, oral dopaminergic medication falls short after a few years and advanced therapies such as deep brain stimulation, continuous intestinal gel levodopa/carbidopa, and subcutaneous apomorphine administration, are considered as alternative options [13]. However, such treatments are administered only in a small subset of patients as they have limited benefit while they are associated with serious side effects, including deterioration of non-motor symptoms [14]. Therefore, although the field of therapies for PD continues to expand, there is still no therapeutic strategy capable to change or reverse the disease course.

Most present-day efforts in identifying novel PD drugs target the aggregation of misfolded α-synuclein (αSyn) as the major pathogenic factor that causes cellular toxicity (reviewed in [15]). αSyn is a presynaptic neuronal protein linked genetically and neuropathologically to PD [16,17,18]. It accumulates abnormally in the PD brain and is the main component of intracellular neuronal inclusions, termed Lewy bodies or Lewy neurites, which represent the histopathological hallmark of PD. The pioneering discovery that αSyn is the major gene associated with both sporadic and genetic PD [16,19,20] spurred intensive research over the past two decades for understanding the mechanisms of αSyn misfolding, oligomerization and aggregation. These studies resulted in deciphering the molecular pathways involved (reviewed in [21]) and developing drugs aiming to halt or even clear αSyn aggregation. A number of promising compounds have been developed targeting pathological αSyn in animal models of PD (reviewed in [22]), but have largely failed when tested in early phase clinical trials, causing disappointment. Several limitations lie behind these unsuccessful endeavors. Notably, despite the flood of studies that have revealed numerous disease-associated processes, fundamental knowledge gaps still exist in PD pathophysiology. Our understanding of the disease covers processes such as mitochondrial impairment resulting in energy failure and increased oxidative stress; lysosomal deficiency and misfolded protein accumulation in large inclusions impeding axonal transport; distorted synaptic vesicle trafficking affecting neurotransmission and synaptic connections. Yet the spatiotemporal sequence of events leading to PD and, most importantly, the initial disease-triggering factors still remain elusive [2,23]. Strikingly, a recent study in which cutting-edge imaging technologies were applied indicated that αSyn immunopositive Lewy bodies and Lewy neurites consist mainly of lipid vesicle clusters instead of the long-assumed proteinacious core [24], pointing to a paradigm shift with important implications for understanding and treating neurodegeneration in PD.

Another drawback in the course of PD drug development is that assessment of novel therapeutics has been performed in animal models that fail by large to recapitulate the human condition. Last, but not least, the traditionally held conviction that the basal ganglia is the main brain region implicated in PD, has been recently challenged. It is now acknowledged that PD is a system-level disorder that affects overlapping neural circuits comprising the entire basal ganglia-cortico-cerebellar axis, which works in concert to mediate motor and cognitive functions [25]. Embracing such a system-level perspective together with using human cell-based disease models for understanding and treating PD has higher potential for the development of innovative treatments.

In this review, we discuss how revolutionary human cell-derived systems based on induced pluripotent stem cell (hiPSC) technology offer an unprecedented opportunity to study PD in models that exhibit clinically relevant disease phenotypes. Such systems provide powerful platforms for understanding pathology and, in combination with recent cutting-edge drug discovery technologies, open up new perspectives for identification of disease-modifying compounds. Yet, there are still limitations and hurdles to face ahead before these young technologies come to fruition and fulfill their promise, particularly in reducing the number of drug failures in clinical trials.

## 2. Identification of Disease-Relevant Phenotypes in hiPSC-Derived Models of PD: A Glimpse into Human Pathology

**Figure 1:**
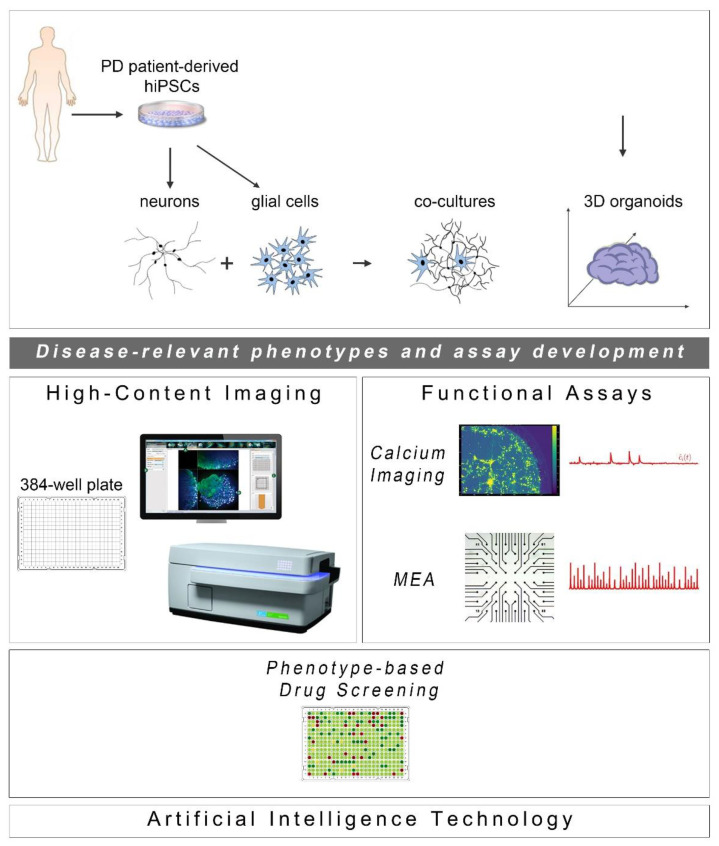
Cell reprogramming technologies allowed the generation of Parkinson’s disease (PD) patient-derived hiPSCs that are further differentiated into neuronal and glial cell populations (astrocytes (shown), but also oligodendrocytes or microglia (not shown)) studied separately or in co-culture with neurons. hiPSC-derived brain organoids are being created to better simulate the human disease. The PD-relevant phenotypes identified in these cellular systems form the foundations for the development of drug discovery platforms encompassing high-content imaging/chemical library screening as well as functional assays, such as calcium imaging and high-resolution multi-electrode array (MEA) recordings (phenotypic drug screening). The application of artificial intelligence technologies will be critical for analysis of the resulting large and complex data sets.

Since the initial discovery of human cell reprogramming technology in 2007 [26], a new exciting era for the field of drug discovery has been ushered. hiPSCs have been widely used to generate “disease-in-a-dish” models for numerous neurodegenerative diseases from which selected cellular systems have been applied in drug evaluation for efficacy and toxicity assessment (Figure 1). Compared to other traditional cellular systems and existing animal models, hiPSC-based platforms offer multiple advantages: human origin in a personalized manner, easy accessibility and expandability, capacity to give rise to many different cell types and avoidance of ethical concerns associated with the use of human embryonic stem cells. Moreover, scalability and manipulation of hiPSC lines for obtaining target cells in large quantities and desirable purity is also possible at GMP grade. In the PD field, intensive efforts have been made to generate high-purity human ventral midbrain dopaminergic neurons for in vitro maturation, but mainly for intracerebral transplantation that has long been an attractive prospect for PD treatment (reviewed in [27,28]). To date, a number of sporadic and familial PD hiPSC-based models have been created displaying a variety of disease-relevant characteristics that could be exploited in either target-based or unbiased phenotypic screens. The percentage of dopamine neurons in these models varies as validated by the expression of markers associated with neuronal type and subtype specificity. However, this may not be a problem, but rather an advantage, in view of the realization that PD is more likely a system-level disorder rather than a dysfunction of the nigrostriatal dopaminergic system.

The first PD-patient specific iPSC lines were generated in 2008 by Park et al. [29] followed by a second group [30]. Both teams provided proof-of-principle that it is feasible to produce hiPSC-derived dopaminergic neurons from patients with sporadic PD, but did not proceed further to identify disease-associated phenotypes. Since then, the majority of hiPSC-based PD models have been developed mostly from patients carrying various genetic mutations (reviewed in [31]). Most studied lines carry the G2019S mutation in the *LRRK2* gene, one of the most prominent monogenetic risk factors for PD linked to both familial and sporadic forms of the disease (reviewed in [32]). hiPSC-derived cultures from patients bearing point mutations in the *SNCA* gene (A53T, A30P and E46K) or *SNCA* multiplications have also been extensively studied, while hiPSC-derived neurons carrying *Parkin* (*PARK2*), *PINK1* or *GBA* mutations have been analyzed to a lesser extent (reviewed in [33]). Overall morphological, biochemical and functional analyses have revealed a battery of disease-associated phenotypes (Table 1), initially after induction of cellular stress and later—upon careful observations—at basal conditions. Some of these phenotypes were previously described in post-mortem PD brains or in relevant animal models, while a number were identified for the first time in hiPSC-based models.

### 2.1. α-Synuclein Accumulation

As discussed, αSyn is the major protein associated with both sporadic and genetic PD [19,20]. It is a small neuronal protein widely distributed in the brain with preferential localization at pre-synaptic terminals and direct association with synaptic vesicles, suggesting possible roles in the regulation of neurotransmitter release, synaptic function and plasticity [21]. Even though the physiological function of αSyn is not well understood [34], its involvement in neurodegeneration is well established [35]. αSyn accumulation and/or aggregation has been shown in hiPSC-derived neurons generated from PD patients carrying disease-associated mutations. In particular, increased αSyn protein levels were detected in hiPSC-derived neurons carrying the A53T (G209A) point mutation [5,36,37,38,39] or *SNCA* duplication [40] and triplication [39,41,42,43,44,45,46]. Accumulation of αSyn has also been described in several other hiPSC models of familial PD, including *LRRK2*, *Parkin*, *PINK1* and hiPSC-derived neurons from *GBA* patients, as well as, in hiPSC models of sporadic PD (reviewed in [33]). Detection of the pathological species relies largely on the presence of phosphorylated αSyn, particularly at serine 129, but also on the presence of specific oligomeric and fibrillar forms as aggregation proceeds. The presence of cellular protein aggregates has also been observed using fluorescence-based assays. In particular, our team [38] and Ryan et al. [36] have shown discrete αSyn accumulations co-localizing with Tau and ubiquitin in both the soma and axons of hiPSC-derived neurons from patients bearing the A53T mutation.

### 2.2. Mitochondrial Defects

Mitochondrial impairment is thought to be a key factor in PD pathology, with PD-associated mutations in *Parkin*, *PINK1*, *DJ-1*, *GBA*, *LRRK2* and *SNCA* being linked to distortions in mitochondrial function [47]. Studies in hiPSC-derived neurons from PD patients have revealed mitochondrial defects, including morphological and functional alterations (reviewed in [33]). In particular, fragmented mitochondria or mitochondria with abnormal morphology have been observed in hiPSC-derived neurons carrying *LRRK2-G2019S* [48], *Parkin* [49,50,51,52,53], *PINK1* [51,54] or *GBA* mutations [55], but also *SNCA-G209A* or triplication [39]. Others have reported decreased mitochondrial content in patient neurons [56,57,58]. Altered mitochondrial functionality has also been demonstrated [52,55,57], with decreased ATP production [43,48], reduced membrane potential [59] or dysfunctional mobility [60], affecting multiple cellular processes and altering the redox status of the neuron.

### 2.3. Oxidative Stress

As mitochondrial respiration is the major source of reactive oxygen species (ROS) in the cell, mitochondrial defects should result in an increase in oxidative stress within patient neurons. A number of studies have demonstrated changes in mitochondrial and oxidative stress-related proteins observed in the human parkinsonian brain (reviewed in [61]). Similarly, oxidative stress phenotypes, such as increased ROS and carbonylated proteins or upregulation of proteins involved in dopamine oxidation, have been observed in several hiPSC-based models carrying *LRRK2* [62,63], *Parkin* [49,50,64] and *PINK1* [60,65] mutations as well as in hiPSC-derived neurons with *SNCA*-triplication [41,43,46].

### 2.4. ER Stress and Autophagy-Related Phenotypes

Consistent with protein aggregation and cellular stress, studies in hiPSC models reveal that endoplasmic reticulum (ER) dysregulation and increased ER stress may be involved in PD pathogenesis promoting neuronal cell death [37,45,66]. Additionally, altered function of other cellular organelles, such as lysosomes, together with autophagy impairment, has been observed in *LRRK2* [48,63,67], *SNCA* [44] and *GBA* [68,69] mutant hiPSC-derived neurons, as well as in a hiPSC-based model of sporadic PD [67], resulting in defective clearance of aggregated proteins. Finally, several studies have shown that genetically diverse PD patient-derived neurons exhibit increased susceptibility to various forms of cellular stress, including proteotoxic [38,62,70] or nitrosative stress [36,37], linking specific cellular pathways to disease pathology that have not been previously described in other systems.

### 2.5. Compromised Neuritic Growth, Axonal Degeneration and Decreased Synaptic Connectivity

Interestingly, studies on *LRRK2-G2019S* [67,71,72,73,74], on *Parkin*-mutated [75] and on *SNCA*-mutated [38,44] hiPSC-derived neurons have revealed previously unrecognized morphological changes, including compromised neuritic outgrowth, reduced neurite complexity and axonal degeneration, resulting in impaired synaptic connectivity and network function. In particular, *LRRK2-G2019S* mutant neurons displayed shorter and less complex processes, reminiscent of immature neurons. Over time, these cells exhibited clear signs of degeneration, including very short or absent neurites, vacuolated soma, fragmented nucleus and positive staining for cleaved caspase-3. In the case of *SNCA* mutations, αSyn overexpression due to triplication of the gene led to poor formation of the neuronal network that correlated with significantly lower generation of action potentials in response to current injections [44]. In a later study performed by our team, A53T-αSyn neurons had a profound downregulation of mRNAs associated with synaptic formation, maintenance and function. The neuronal network formed was also less complex with neurite number, length and morphology being compromised when compared to control cells [38]. It will be interesting to investigate further whether the impaired synaptic connectivity and aberrant electrophysiological recordings noted in PD neurons represent early events in disease pathogenesis and how they are linked to distorted mitochondrial and/or axonal transport and ER stress. In any case it is an important finding that should be taken into account when developing new therapeutic or disease-intervening strategies.

**Table 1:** Major phenotypes observed in PD hiPSC-derived neurons. This table summarizes key pathogenic phenotypes associated with PD mutant neurons as described in the cited publications.

Major PD-Relevant Phenotypes	Patient-Derived iPSC-Based Models in PD					
	*Gene Mutations*					
	*SNCA*	*LRKK2*	*PARKIN*	*PINK1*	*GBA*	*OPA1*
αSyn accumulation and/or aggregation; increased phosphorylated αSyn (Ser 129); presence of oligomeric and fibrillar αSyn forms	G209A [5,36,37,38,39] Duplication [40] Triplication [39,41,42,43,44,45,46]	G2019S [62,67,71,72,73]	Ex2–4 del and Ex6–7 del [49] Ex3del, R42P, Ex3–4del, 1-BP del 255A, R275W, R42P [56] V324A [51] c.255delA [72]	Q456X [51]	L444P [68] N370S [69]	
Mitochondrial defects: fragmented mitochondria or mitochondria with abnormal morphology; decreased mitochondrial content; decreased ATP production; reduced membrane potential; dysfunctional mitochondrial mobility	G209A Triplication [39]	G2019S [48,57]	Ex2–4 del and Ex6–7 del [49] Ex3, 5, and 6 del [50] V324A [51] c.1072delT, p.A324fsX110 [52] Ex2–4 del and Ex6–7 del [53]	Q456X [51] G309D; Ex7/del [54]	N370S, L444P, and RecNcil [55]	p.G488R and p.A495V [58]
Oxidative stress: increased ROS and carbonylated proteins; upregulation of proteins involved in dopamine oxidation	Triplication [41,43,46]	G2019S [62] I2020T [63]	Ex2–4 del & Ex6–7 del [49] Ex3, 5, and 6 del [50] Ex3 del/Ex5 del and Ex3 del/Ex 3 del [64]	Q456X [60,65]		
ER dysregulation; increased ER stress; autophagy impairment	G209A [37,66] Triplication [44,45]	G2019S [48,67] I2020T [63]			RecNcil, L444P and N370S [68] N370S [69]	
Compromised neurite growth & complexity; neurite swellings; axonal degeneration; decreased synaptic connectivity; impaired axonal transport	G209A [38] Triplication [44]	G2019S [67,71,72,73]	Ex3 del/Ex5 del and Ex3 del/Ex 3 del [75]			

## 3. Rescue of Disease-Related Phenotypes in hiPSC-Derived Models of PD: Setting the Foundations towards Drug Discovery

Contrary to the initial skepticism regarding the potential of hiPSC-based systems to recapitulate age-related diseases such as PD, the identification of a wealth of cellular and molecular phenotypes, either previously known or novel, has prompted the use of such systems for early drug discovery. A number of candidate drugs have been tested for their ability to restore disease-related phenotypes in hiPSC-derived models of PD with promising results (Table 2). These studies have laid the groundwork for future application of hiPSC models in larger drug screening campaigns, assisting in the development of robust assays.

Analyses of hiPSC-derived neurons from patients with different PD-linked mutations have shown that αSyn accumulation presents a common theme regardless of the gene mutated, suggesting that it could serve as a read-out when testing novel therapeutics. Accordingly, in hiPSC-derived dopaminergic neurons from sporadic PD patients, as well as from patients carrying mutations in *GBA*, *LRRK2*, *DJ-1*, *Parkin* and *SNCA* (A53T, triplication) with decreased lysosomal β-glucocerebrosidase (GCase) activity, treatment with small-molecule enhancers of GCase has resulted in reduced αSyn levels and associated toxicity. These observations suggest that non-inhibitory small-molecule chaperones of GCase may prove promising for treatment of PD and related synucleinopathies [76,77,78]. Additionally, recent data in *GBA*-linked PD patient-derived dopaminergic neurons have implicated increased acid ceramidase activity in the context of decreased GCase. This led to intracellular accumulation of αSyn, the levels of which were reduced upon inhibition of acid ceramidase by carmofur [79]. In the same lines, Burbulla et al. have applied treatment with mitochondrial antioxidants to smooth out a pathological cascade instigated by mitochondrial oxidant stress causing lysosomal dysfunction and αSyn accumulation in dopaminergic neurons derived from patients with sporadic and familial PD [80].

Alterations in neuronal morphology, neurite outgrowth and complexity as well as axonal degeneration have been used as parameters to evaluate the potential efficacy of compounds in rescuing pathological phenotypes. One study has demonstrated that *LRRK2-G2019S* hiPSC-derived neurons display neurite shortening and fewer neurites compared to wild-type controls [71], a PD-associated phenotype previously linked to ERK signaling [81]. Interestingly, treatment with an inhibitor of ERK phosphorylation or a selective inhibitor of LRRK2 kinase activity increased neurite growth and rescued mutant cultures from degeneration [71]. Similarly, Korecka et al. demonstrated that exposure to a LRRK2 kinase inhibitor or application of a *LRRK2*-specific antisense oligonucleotide (ASO) rescued neurite collapse, also in a model of *LRRK2-G2019S* hiPSC-derived neurons [74].

In the patient-derived *SNCA-G209A* (A53T) hiPSC-based model that we developed, PD neurons exhibited distinct morphological features characterized by extensive neuritic pathology and degeneration [38]. By immunostaining for βIII-tubulin, we observed contorted or fragmented axons with swollen varicosities and spheroid inclusions containing Tau and αSyn. Under morphological examination using a lentiviral vector for expression of the red fluorescent protein DsRed under the control of human synapsin 1 promoter (LV. SYN1.DsRed) to facilitate imaging of single neurons, we also observed a significant reduction in both total neurite length and the number of neurites extending from the soma. Three de novo in silico-designed compounds [82,83] that all interact with and reduce αSyn toxicity by interfering with αSyn oligomer formation could restore neurite length and rescue axonal pathology [38]. Interestingly, a small variation of one of these small molecules, NPT200-11, that was developed by the company Neuropore Therapies in collaboration with UCB Biopharma, is the only αSyn-inhibiting compound that has reached clinical trials successfully completing phase I (https://clinicaltrials.gov/ct2/results?cond=&term=NPT200-11&cntry=&state=&city=&dist=, Study Completion Date: February 2016).

Several studies with hiPSC-derived neurons have revealed dysregulation in the expression of genes involved in numerous cellular processes, rendering cells vulnerable to stressors that activate or modulate these pathways. Oxidative stress, mitochondrial impairment and proteasome inhibition are key factors that cause increased susceptibility and cell death of patient-derived neurons (reviewed in [33,84]). The first study recapitulating PD-associated phenotypes has revealed that dopaminergic neurons derived from *LRRK2-G2019S* hiPSCs displayed increased expression of key oxidative stress-response genes. Moreover, these cells were highly sensitive to cell death caused by exposure to hydrogen peroxide, the proteosomal inhibitor MG-132 or the neurotoxin 6-hydroxydopamine (6-OHDA) [62]. In a similar manner, Reinhardt et al. have confirmed that *LRRK2-G2019S* hiPSC-derived dopaminergic neurons are susceptible to oxidative stress induced by the mitochondrial complex 1 inhibitor rotenone or the neurotoxin 6-OHDA resulting in increased apoptosis, preferentially of dopaminergic neurons [71]. The incurred cytotoxicity was rescued in the presence of the small molecule inhibitor of LRRK2 kinase, LRRK2-IN1, which increased the survival of dopaminergic neurons.

An association between PD and exposure to mitochondrial toxins, including rotenone, has also been reported in *SNCA-G209A* (A53T) mutant neurons [36]. Microarray analysis of *SNCA-G209A* hiPSC-derived dopaminergic neurons has highlighted a pathway where toxin-induced nitrosative/oxidative stress results in S-nitrosylation of the transcription factor MEF2C. High-throughput screening of a chemical library for small molecules capable of targeting the MEF2C-PGC1α pathway pinpointed isoxazole as a potential therapeutic, protecting *SNCA-G209A* neurons from apoptosis induced by mitochondrial toxins [36].

When exposing the A53T-αSyn hiPSC-based model that we generated to epoxomicin or MG132, both of which interfere with αSyn clearance via the proteasome, we observed a significant increase in cleaved caspase-3 immunoreactivity consistent with the levels of LDH release in mutant neurons. This was accompanied by a pronounced disruption of the MAP2+ neuronal network, confirming once again their susceptibility to proteotoxic stress [38]. The observed stress-induced vulnerability could also be reversed by the three small molecules targeting αSyn [82,83], resulting in restoration of the MAP2+ network [38].

Finally, a recent study has revealed that increased oxidative stress and inflammation are associated with induction of the necroptotic pathway in hiPSC-derived neural cells from patients with a mutation in the *OPA1* gene encoding a key player in mitochondrial fusion and structure [58] that has been associated with an inherited form of PD and dementia [85]. Mutant cultures exhibited severe mitochondrial dysfunctions, impaired oxidative phosphorylation, and high oxidative stress levels, leading to neuronal cell loss. Pharmacological treatment of necroptosis with the specific inhibitor necrostatin-1 protected neurons from cell death [58].

The above paradigms provide evidence that, despite the initial concerns in using human iPSC-based models for modeling age-related neurodegenerative pathologies, such systems show multiple disease-associated phenotypes with high relevance to PD pathogenesis and progression and can be of great value in drug discovery.

## 4. Phenotypic Screens using hiPSC-Derived Models of PD: Empowering Drug Discovery

### 4.1. Target-Based Versus Phenotype-Based Drug Screening

The two main high-throughput screening approaches for discovering new disease-modifying therapeutics are either target-based or phenotype-based. Historically, phenotypic-based screening strategies shaped the foundations of pharmaceutical drug discovery long before molecular target-based approaches were applied [86]. However, in the past 25 years, molecular target-based drug screening has become the main route to drug discovery in both the academia and the pharmaceutical industry. This change was mainly due to an accelerated progress in molecular biology and genomics that resulted in efficient mining of genes associated with various diseases [87]. The starting point in this approach is a well-defined molecular target with a predicted role in disease allowing the hypothesis that modulation of its activity would have beneficial effects. Screening of chemical libraries of small molecules is then used to identify lead compounds that interact with high affinity and specificity with the target [88]. Hits from such screens are then used for pharmacological target validation and lead compound optimization. The main advantage of the target-based approach is that the mechanism of action is known right from the start, which can accelerate preclinical assessment. Other advantages include the ability to facilitate optimization of the lead compound as there is a clear structure–activity relationship enabling improvement of its physicochemical properties, and the potential to predict target-associated safety liabilities and toxicity. However, knowledge of the molecular targets has not translated into identification of disease-modifying agents for PD or other neurodegenerative diseases [89]. One reason could be that the underlying mechanism is not clear for PD, as for most neurodegenerative diseases, resulting in a universal lack of well-defined targets. Besides, neurodegenerative diseases, including PD, are highly complex disorders and manipulating a single target may not be sufficient to restore the dysfunctional cellular network.

In phenotypic screens, on the other hand, disease-driving phenotypes can be used to determine compounds that change the outcome of multiple biological pathways without prior knowledge of the molecular mechanisms of the disease. Such screens are unbiased and may identify compounds targeting completely unexpected proteins or pathways. Consequently, phenotypic screens hold promise for the identification of previously unrecognized disease pathways and the discovery of new therapeutic targets [90]. It is notable that, during the past 20 years, phenotypic screening has contributed to most of the first-in-class small-molecule drugs approved by the FDA. Among all the new molecular entities approved from 1999 to 2008, 28 were identified through phenotypic screens, whereas target-based approaches contributed to the discovery of 17 compounds [91]. In particular, in the central nervous system field, phenotypic screening has yielded seven out of nine first-in-class drugs. Consequently, there is renewed interest in reinventing phenotypic screens as a means of drug discovery.

### 4.2. Phenotypic-Based Drug Screening in hiPSC-Derived Models of PD

Although the first PD patient-derived hiPSCs were generated in 2009 [30], surprisingly only two phenotypic screens have been reported so far in hiPSC-derived PD neurons. To identify disease-modifying agents, Yamaguchi et al. established an imaging-based, semi-automatic, high-throughput assay for quantitative detection of mitochondrial clearance and cell viability in dopaminergic neurons from patients with familial PD having *Parkin* or *PINK1* mutations. After screening 320 pharmacologically active inhibitor compounds, the researchers identified four hits, MRS1220, tranylcypromine, flunarizine and bromocriptine, that improved the pathological clearance of mitochondria possibly by promoting mitochondrial degradation through the lysosomal system, without further investigating the underlying mechanism [92]. In another study, Tabata et al. [93] performed a phenotypic screen in *Parkin* (*PARK2*) patient-derived dopaminergic neurons displaying increased susceptibility to rotenone-induced mitochondrial stress, to identify neuroprotective compounds. From phenotypic screening of an FDA-approved drug library, one voltage-gated calcium channel antagonist, benidipine, was found to suppress rotenone-induced apoptosis [93]. The selective vulnerability of dopaminergic neurons was further attributed in this study to the dysregulation of intracellular calcium homeostasis via T-type calcium channels, revealing a previously unidentified pathway in PD and offering a potential treatment opportunity. More recently, using A53T-αSyn patient-derived neurons, we performed a small-scale phenotypic screen to identify neuroprotective compounds and identified the multikinase inhibitor BX795 as a candidate therapeutic that rescued the pathological features of PD neurons [94].

## 5. Looking into the Future: Optimization of hiPSC-Based Models for Understanding and Treating PD

The development of phenotypic screens in PD hiPSC-based neuronal cultures is still underway with the field awaiting the identification of novel disease targets for therapeutic interventions. In the meantime, new scientific discoveries and technical advances underscore the limitations of using two-dimensional (2D) neuronal platforms lacking a more physiological environment, such as extracellular matrix and the presence of glial cells. Emerging developments highlight the enhanced potential of using neuronal and glial co-cultures as well as three-dimensional (3D) systems of various kinds, including brain organoids that simulate more closely the human pathophysiology. Current progress in these approaches complemented by advances in microfluidics, state-of-the-art imaging methodologies and electrophysiological analyses to evaluate the functionality of neuronal networks, is discussed below (Figure 1).

### 5.1. Glial Cell Involvement in PD Pathogenesis: Mimicking the CNS Microenvironment in hiPSC-Based Co-Culture Systems

As for most neurodegenerative diseases, the majority of PD studies have been performed in neuronal cultures, preferably consisting of midbrain dopaminergic neurons in various degrees of purity, and assessing neuronal degeneration signs after exposure, or not, to different types of stress. However, neuronal death may also be induced by a microenvironment that does not sufficiently support neuronal survival and/or function, while newer concepts suggest that other neural cell types, such as astrocytes [95] and microglia [96], may contribute to PD pathogenesis and progression.

Studies in post-mortem human brains and in animal models of PD, including Parkinsonian macaques [97,98,99,100,101], have demonstrated astroglial activation, suggesting that astrogliosis in combination with the secretion of pro-inflammatory cytokines may contribute to PD progression [102,103,104]. Moreover, abnormal αSyn accumulation has been observed in post-mortem astrocytes indicative of pathological alterations [101]. One explanation that has been provided is that neurons transfer pathological αSyn to astrocytes, which in turn could have an active role in its clearance [105]. It is therefore possible that astrocytes could have either a detrimental or a beneficial role, or both depending on the disease stage. It is plausible to assume that, at early stages of PD, astrocytes act to protect neurons from an overload of pathological protein cargo, while, at later stages, they become dysfunctional and contribute to the deterioration of neuronal health.

As evidenced by positron emission tomography (PET), microgliosis is an early and sustained response in PD [106,107], while reactive microglia have also been detected in toxin-induced or transgenic mouse models of PD [108,109,110,111]. Interestingly, various studies support that microglia-mediated inflammation can have both beneficial and detrimental effects [112,113,114,115,116,117]. For example, the interaction of pathogenic αSyn with different microglial receptors promotes microglial clearance of αSyn and phagocytosis of apoptotic neurons, which may be beneficial in controlling pathology [118,119,120,121]. On the other hand, it has been shown that neurotoxic microglia can induce the generation of A1-type astrocytes exhibiting a neurotoxic phenotype, thus revealing a deleterious effect of microglial activation [122]. Interestingly, blocking the microglial-induced A1 astrocyte conversion has been found to be neuroprotective in models of PD offering new therapeutic prospects [123].

Oligodendrocytes appear to be the least affected cell type in PD, although they too present αSyn depositions [101] and show intrinsic formation of pathological αSyn assemblies [124]. Intriguingly, a reduction in the myelination of neuron projections seen in the earliest stages of the disease provides a possible association between oligodendrocyte function and PD pathogenesis [125].

Human cell-derived in vitro models can provide more specific information on the positive and/or negative involvement of glial cells in PD pathogenesis and progression. So far, only a few studies have been reported that explore hiPSC-derived systems for investigating the astrocytic involvement in PD. Human midbrain astrocytes generated from PD patients carrying the *LRRK2-G2019S* mutation showed transcriptomic dysregulation associated with compromised ability to degrade αSyn [126]. A more distinctive involvement of hiPSC-derived astrocytes transpired when they were co-cultured with dopaminergic neurons. In particular, when co-cultured with *LRRK2-G2019S* astrocytes, control hiPSC-derived dopaminergic neurons acquired morphological signs of neurodegeneration and abnormal, astrocyte-derived αSyn accumulation. Conversely, control astrocytes partially prevented the appearance of disease-related phenotypes in PD neurons [127]. In a recent study, astrocytes derived from patients with *GBA*-associated Parkinsonism displayed a cytokine and chemokine profile indicative of an inflammatory response [128]. Moreover, when these cells were co-cultured with dopaminergic neurons generated from the same hiPSC lines, excessive αSyn released from neurons was endocytosed by the *GBA*-derived astrocytes, translocating into lysosomes. It therefore seems that in *GBA*-associated Parkinsonism, astrocytes play a role in αSyn accumulation and processing, contributing to neuroinflammation. Similarly, Sonninen et al. have just published that *LRRK2-G2019S* hiPSC-derived astrocytes exhibit pathological hallmarks of the disease, including increased αSyn expression, which results in altered metabolism, disturbed Ca2+ homeostasis and increased release of cytokines upon inflammatory stimulation [129]. These findings are in line with the manifestation of αSyn inclusions in activated astrocytes in the post-mortem human PD brain as well as in animal models and suggest that pathogenic astrocytes may contribute to non-cell autonomous build-up of toxic αSyn species and the initiation of neuronal deterioration.

Recent developments in more efficient methodologies have allowed for the generation of hiPSC-derived macrophages that can be induced towards a microglial phenotype by co-culture with neurons [130]. These derived microglial cells acquired a highly dynamic ramified morphology and exhibited neuronal surveillance activity mimicking the in vivo situation. Building on this advancement, a subsequent study demonstrated that *LRRK2* expression in macrophages and microglia plays an important role in phagosome maturation and in the regulation of recycling pathways, implying that *LRRK2* mutations in PD patients may disrupt microglial clearance mechanisms [131]. Additionally, it has been shown that the *LRRK2-G2019S* mutation influences fate decision in hiPSC-derived human monocytes, further endorsing the involvement of the immune system in the development of PD [132].

Overall, the limited research performed so far on more complex human experimental set-ups has emphasized that considering bilateral or non-cell autonomous interactions between neurons, astrocytes and microglia is critical for elucidating the pathogenic processes occurring in PD and uncovering the molecular mechanisms triggering disease appearance and progression. Furthermore, the few studies performed indicated the need for using more complex systems as physiologically relevant disease models for drug discovery.

### 5.2. Emergence of Three-Dimensional (3D) hiPSC- Based Platforms for PD

The appearance of 3D systems of increasing complexity mimicking the brain microenvironment meets the requirement for more efficient modeling of disease phenotypes not only towards elucidating context-dependent human pathologies, but also for the development of novel platforms for high-throughput drug screening. Yet, there are still serious hurdles to overcome, including the reduced long-term viability of such systems due to lack of sufficient oxygenation within the 3D core and the large variability observed [133]. In a first attempt within the PD field, the culture of patient neurons derived from hiPSCs carrying the *LRRK2-G2019S* mutation was optimized in 3D microfluidics [134]. Automated high-content imaging revealed decreased dopaminergic differentiation and branching complexity, altered mitochondrial morphology, and increased cell death in the absence of external stressors. In two subsequent efforts, midbrain-like organoids were produced from sporadic or familial PD patients carrying the *LRRK2-G2019S* mutation that, respectively, displayed αSyn accumulation and dopaminergic neuron degeneration [135,136].

Clearly, further optimization and analysis are needed for the production of advanced patient-specific platforms that can be useful in modelling and treating PD. In this respect, research is progressing fast to generate more complex 3D cultures yielding organoids or spheroids that incorporate neurons and different glial cell types [137,138,139,140,141] offering promising tools to simulate PD pathology with higher fidelity to the in vivo scenario. Enriched 3D systems can be more informative on the cellular and molecular basis of neuron-glia cross-talk, monitoring glial activation and inflammation, also in correlation with toxic αSyn accumulation and neuronal decline. Of relevance, an optimized protocol was devised recently for the generation of midbrain-like organoids containing mature midbrain dopaminergic and GABAergic neurons, functional astrocytes and oligodendrocytes, exhibiting electrophysiological activity and producing dopamine and neuromelanin-like granules [142]. A PD-mimicking neurotoxin-based protocol was then applied to assess cell-to-cell interactions in neurodegeneration, demonstrating glia-mediated massive cell death of dopaminergic neurons. Interestingly, Ormel et al. showed that microglia can also develop innately within cerebral organoids and display their characteristic ramified morphology [141]. This was an unexpected finding since the consensus was that cerebral organoids consist of cells derived from the neuroectodermal lineage and should therefore lack mesodermal-derived microglia. Yet, this study exemplified a model where the interplay between microglia, macroglia and neurons can be studied in human brain development and disease.

Even though 3D organoids have not been used yet for high-throughput drug screening in PD, the technology for adaptation is developing and has been used successfully in other neurodegenerative diseases, indicating the feasibility of such strategies [143]. Developments include a number of microtissue and nanoculture products to support 3D architecture in 96- or 384-well format, introduction of enriched extracellular matrix (ECM) products and inducers of endogenous ECM proteins and specialized synthetic biomaterials [144]. Nevertheless, the high cost of such products, the disruption of viscosity and temperature during automated handling, the sample processing for high-content analysis and the poor penetration of tested compounds present serious limitations for performing preclinical testing in a large scale and within a reasonable timescale. New approaches that target the issues of high-throughput scale and cost while offering amenable systems with a high level of biological complexity and clinical relevance, such as formulation and optimization of engineered microenvironments, are in great demand. Such brain-on-a-chip platforms comprising miniaturized microfluidic perfusion systems that permit long-term growth in a format that is financially viable and has the potential of scaling up for launching high-throughput discovery campaigns, might pave the way for future fundamental discoveries and the development of more effective drugs [145]. Microfluidic devices have already been used to study the interaction of microglia and neurons in PD and to demonstrate neuronal internalization of αSyn fibrils before their propagation along the axons [146,147,148]. However, today, the most relevant formats are restricted to low-throughput applications awaiting their adaptation for automated screening on a large scale.

## 6. Functional Assays for PD Studies and Drug Screening in hiPSC-Derived Systems

As the ultimate success of drug discovery depends on the functional recovery it can produce, a pipeline based solely on assessment of morphological rescue of human PD neurons will have a high chance of failing. PD is characterized by severe changes in neuronal connectivity and data derived from electrophysiological analyses of patient-derived neurons highlight the importance of recording changes in synaptogenesis, network formation and neurotransmitter balance when new drugs are tested or during unbiased phenotypic screens. Two functional assays may be used in drug screening based on their potential for adaptation and scalability: calcium imaging and the multi-electrode array system (Figure 1).

### 6.1. Calcium Imaging

Calcium homeostasis is fundamental to neuronal survival and function and, when deregulated, can lead to neurodegeneration via complex and diverse mechanisms resulting in selective neuronal impairment and death. Fluorescent calcium imaging is a well-established method, which enables the visualization of free intracellular Ca2+ in populations of cells. Calcium indicators are sensitive to calcium changes and can be loaded in a non-invasive manner to neuronal cells, although prolonged exposure to the dye is toxic. Fluorescent dyes can either be single or multiple wavelengths. Calcium imaging allows to explore calcium-mediated processes happening over different time scales. For example, calcium-mediated neurotransmitter release occurs much more rapidly than calcium-mediated gene expression in the nucleus [149]. The development of genetically encoded calcium indicators offers a better alternative to the use of fluorescent dyes, since they are non-invasive and can be targeted to specific neurons and/or astrocytes, allowing for a longer duration of imaging without the risk of phototoxicity [150]. Additionally, other issues observed with fluorescent dyes such as background fluorescence and non-specific dye loading can be overcome with genetic indicators [150].

In relation to PD, increasing evidence suggests that defective calcium signaling plays an important role in disease pathogenesis. Schwab and Ebert showed that *LRRK2-G2019S* hiPSC-derived sensory neurons display altered calcium dynamics and treatment with LRRK2 kinase inhibitors resulted in significant rescue [151]. Moreover, Tabata et al. demonstrated that there is a dysregulation of calcium homeostasis in *Parkin* and *PINK1* hiPSC-derived dopamine neurons which is prevented by T-type calcium channel knockdown or antagonists [93]. We have also demonstrated aberrant Ca+ fluxes in A53T-αSyn neurons [152] that could be linked to the decreased spontaneous synaptic activity recorded by patch-clamp electrophysiology [38]. Currently, high-content imaging or drug screening based on recording Ca+ dynamics has not yet been performed in PD hiPSC-derived systems.

### 6.2. Multi-Electrode Arrays (MEAs)

High-resolution MEA systems enable one to assess novel electrophysiological parameters of hiPSC-derived neurons, which can be potentially used as biomarkers for phenotype screening and drug testing. MEAs comprise a platform for monitoring prolonged, non-destructive recordings of spontaneously firing neurons in vitro with applications in neurodegenerative diseases. A MEA system combines a cell culture plate with an embedded array of high- or low-impedance electrodes allowing for parallel detection of local field potentials generated by spontaneous or evoked firing of neurons [153,154,155]. The generation of synchronously active neuronal networks depends on sequential developmental processes. Neuronal network formation starts with excitable and spontaneously active neurons that are asynchronously active due to lack of functional connectivity between neurons. With the formation of functional synapses, two or more neurons become functionally interconnected and capable to generate synchronous bursting. In a population of neurons, the connectivity between neurons is increasing over time and finally results in synchronous bursting activity of hundreds or thousands of interconnected cells. So, calcium imaging and MEA recordings could reveal the inability of pathological cells to make the physiological transition of asynchronously active neurons into few synchronous bursting neurons and finally into a population of neurons that are highly synchronously active. One characteristic example is described by Woodard et al., in which hiPSC-derived dopamine neurons were examined from monozygotic male twins of Ashkenazi Jewish background that were discordant for PD [156]. The investigators noticed that neurons from the unaffected twin developed robust synchronous bursting patterns indicative of maturing neuronal networks in contrast to neurons from the affected twin that did not produce synchronous bursting patterns with their spontaneous activity also being significantly lower [156]. In another study, however, on hiPSC-derived dopaminergic neurons from patients with young-onset PD, MEA recordings did not show differences between disease and control cultures [157].

Nonetheless, the value of MEA recordings in uncovering disease-related phenotypes in an in vitro setting is highlighted in primary cultures derived from animal models of neurodegenerative diseases. Amin et al. applied MEA recordings to characterize the early activity-dependent changes induced by toxic Aβ-oligomers in neuronal networks using a simple in vitro model based on a rat hippocampal cell culture system. It is interesting to note that, in this study, a clinically applied N-methyl D-aspartate antagonist used for Alzheimer’s disease treatment, could reverse Aβ-neurotoxicity and rescue network-wide firing [158]. The importance of monitoring coordinated neuronal activity and its disruption in the development of neurodegeneration has also been emphasized by Iaccarino et al. in a mouse model of Alzheimer’s disease [159].

Regarding the adaptation of the methodology for use in high-throughput screening, Durens et al. have described a method for multiplexing MEA recordings and Ca2+ imaging to examine local microcircuits in 3D brain organoids and assess inter-experimental consistency, necessary for drug screening [160]. Examination of such circuit-based mechanisms in hiPSC-derived systems would be of great interest and importance for the development of new and more efficient drugs. However, while it seems a very attractive readout for the development of high-throughput drug screening platforms, one should be careful with the interpretation of MEA and calcium imaging results as chemically induced effects on neuronal network activity, such as spontaneous firing and bursting behavior, depend on the ratio of inhibitory to excitatory neurons that are present in the culture system and may vary either due to the differentiation and enrichment protocols applied or to intrinsic defects of the lines used. Therefore, hiPSC-derived neuronal models must be carefully characterized prior to large-scale functional applications in drug screening.

## 7. Artificial Intelligence Technologies

Concurrent with advances in imaging technologies and the morphological, biochemical or functional analyses of neuronal populations has been the increase in volume and complexity of data sets generated. To exploit the ever-growing amount of data that become available, computational techniques are constantly evolving. As a result, artificial intelligence technologies such as deep learning and machine learning methods have emerged to identify meaningful patterns and cellular features that may be interpreted into novel biological insights. Still, these approaches have yet to be developed and adapted for high-throughput analyses in the medical and biological sciences. The creation of automated platforms that can perform phenotypic screens, process raw data and analyze them using deep neural network algorithms will increase both the capacity of the screens as well as the quality and size of the data collected. Several groups are trying to develop new methods that would have the potential to be used in large-scale phenotypic screens of hiPSC-based models. For example, Schwartz et al. developed a 3D model of hiPSC-derived neural tissue constructs comprising diverse neuronal and glial populations, interconnected vascular networks, and ramified microglia by seeding neural precursor cells on synthetic hydrogels [161]. Machine learning was used to build a predictive model from the changes occurring in global gene expression resulting from exposure to toxic and nontoxic training chemicals. This combined strategy reveals the value of human cell-based assays for predictive toxicology. Similarly, Monzel et al. developed a pipeline for a machine learning method, allowing for detailed image-based cell profiling and toxicity prediction in brain organoids treated with the neurotoxic compound 6-hydroxydopamine (6-OHDA) [162].

The analysis of large biological data sets derived from high-throughput screens assessing simultaneously multiple morphological and functional parameters in hiPSC-based platforms is time-consuming and will certainly benefit from artificial intelligence methodologies. Machine learning has the potential to shrink drug discovery timelines, helping researchers accelerate drug discovery and ultimately patients obtain disease-modifying therapies. Because these methodologies are expected to make the quest for new pharmaceuticals not only quicker, but also cheaper and more effective, leading biopharmaceutical companies to begin to embrace artificial intelligence platforms in their pursuit for new therapies. However, in the short term, these technologies have a number of challenges to overcome, especially when combined with automation.

## 8. Concluding Remarks

Many hiPSC lines have been generated from patients with familial or sporadic PD uncovering known or previously unrecognized disease-relevant phenotypes that, in many cases, could be effectively restored using small molecules. These investigations have laid the foreground for developing bioassays for screening small or larger chemical libraries in the search of lead compounds that may evolve into PD disease-modifying therapeutics. However, robust assays still need to be established before hiPSC-based systems become amenable to high-throughput technologies. In the meantime, more advanced co-culture systems encompassing neurons and glial cells or 3D brain organoids mimicking more closely the in vivo human situation are being developed to assist in PD studies and drug discovery. The necessity for functional assays to predict drug efficacy is also being recognized while technological advancements render complicated screens more feasible. Last but not least, the emergence of artificial intelligence over the past few years may prove to be a game-changing technology in drug discovery. Nevertheless, opportunities and challenges still remain ahead before these young technologies come to fruition and fulfill their promise for understanding and treating neurodegeneration in PD.

## Figures and Tables

**Table 2:** Summary of hiPSC-based models of PD that have been used for drug testing. This table briefly describes the rescue of disease-related phenotypes in PD hiPSC-derived neurons by selected compounds.

Gene	Phenotypes Described	Compound Testing	Phenotype Restored	Reference
*GBA* mutations	increased levels of αSyn; reduced lysosomal GCase levels, reduced lysosomal GCase activity	small-molecule noninhibitory chaperone of GCase NCGC607	reduced αSyn levels and associated toxicity	[76]
*GBA* (N370S/c.84dupG), *SNCA*- triplication	presence of amyloidogenic αSyn within cell bodies and neurites; accumulation of insoluble αSyn; reduced neuronal viability; reduced lysosomal activity of GCase	small-molecule GCase modulator 758	improved GCase activity; reduced αSyn levels	[77]
*GBA* (c.84dupG frameshift mutation) *LRRK2* and *Parkin* mutations	reduced amounts of GCase; decreased GCase enzymatic activity; accumulation of oxidized dopamine	small-molecule GCase modulator S-181	increased amounts of lysosomal GCase; enhanced GCase enzymatic activity; decreased dopamine oxidation	[78]
*GBA* (c.84dupG frameshift mutation)	increased acid ceramidase activity in the context of decreased GCase, leading to intracellular accumulation of αSyn	carmofur, acid ceramidase inhibitor	reduced αSyn levels	[79]
*DJ-1* mutations	mitochondrial oxidant stress causing lysosomal dysfunction and αSyn accumulation	mitochondrial antioxidants	diminished accumulation of oxidized dopamine; improved lysosomal GCase activity and proteolysis	[80]
*LRRK2* (G2019S)	reduced neurite outgrowth; increased sensitivity to oxidative stress	ERK phosphorylation inhibitor PD0325901 or LRRK2 kinase inhibitor LRRK2-IN1	increased neurite growth; reduced cytotoxicity	[71]
*LRRK2* (G2019S)	neurite collapse; altered ER calcium homeostasis	LRRK2 kinase inhibitor Mli-2 or *LRRK2*-ASO	rescued neurite collapse	[74]
*SNCA* (G209A)	αSyn aggregation; mitochondrial dysfunction; increased susceptibility to mitochondrial toxins	small molecule targeting MEF2C-PGC1α (isoxazole)	reduced apoptosis	[36]
*SNCA* (G209A)	αSyn aggregation; compromised neurite outgrowth and axonal neuropathology; defective synaptic connectivity	small molecules targeting αSyn (NPT100-18A, NPT100-14A or ELN484228)	improved neurite outgrowth; rescue of axonal pathology and morphological restoration of the neuronal network	[38]
*OPA1* mutation	mitochondrial dysfunction; impaired oxidative phosphorylation and high oxidative stress levels leading to neuronal cell loss	necrostatin-1, specific necroptosis inhibitor	increased survival	[58]

## Abbreviations

PD	Parkinson’s disease
*SNCA (PARK1/4)*	α-synuclein gene
*LRRK2 (PARK8)*	leucine-rich repeat kinase 2 gene
*PINK1 (PARK6)*	PTEN-induced putative kinase 1
*DJ-1 (PARK7)*	protein deglycase
*ATP13A2 (PARK9)*	endo-/lysosomal-associated P5 type transport ATPase
*GBA*	β-glucocerebrosidase gene
αSyn	α-synuclein protein
hiPSC	human induced pluripotent stem cell
GMP	good manufacturing practices
ROS	reactive oxygen species
ER	endoplasmic reticulum
GCase	β-glucocerebrosidase
ASO	antisense oligonucleotide
6-OHDA	6-hydroxydopamine
LDH	lactate dehydrogenase
OPA1	mitochondrial dynamin-like GTPase
PET	positron emission tomography
ECM	extracellular matrix
MEA	multi-electrode array
